# The Sufferings of Christ

**Published:** 1887-08-13

**Authors:** 


### WORDS OF CONSOLATION.

[Communications for this column should be addressed, "Chaplain," care of the Editor of THE HOSPITAL, who will gratefully welcome any suggestions or inquiries from matrons, nurses, and the staff generally.]

### XLV.—THE SUFFERINGS OF CHRIST.

(*For Reading to the Sick.*)

WE all know that our Lord Jesus "Christ suffered for us, leaving us an example, that ye should follow his steps" (1 PET. ii, 21). But what does this knowledge lead to? Does it lead to any deep sympathy with Him? Does it lead, or has it ever led, to any distress on our part for having caused His sufferings? You have been, perhaps, grateful at the very thought of being a redeemed soul. But have you ever been very sorrowful as well at having been personally one of the causes of His Agony, Passion, and Death? "I, if I be lifted up from the earth, will draw all men unto me" (JOHN xii, 32). But we can only be drawn by love, by sympathy, by taking to heart the absolute necessity, and yet willing devotion, of the great sacrifice to our own eternal salvation. He suffered for all. Yes, but for you individually were the fountains of the great deep of Divine love and sorrow broken up, and poured forth, with the same willingness as if you were the only soul to be saved. We can none of us learn to be grateful, we can never hate our sins sufficiently to be rid of their dominion, until we realise the individual share we have had in crucifying the Lord of Glory. This is the knowledge which softens the heart; this is the acceptation of Christ Crucified by the inner man, which makes the Story of the Cross something far transcending in its importance any other fact in the history of the world. You may consider now for a moment just the outline of the Divine love for you which led to the Sacrifice on Calvary. That love for your soul *never had any beginning.* You had a beginning: but God's love for you had not. He loved you in prospect before He created you. He says in one place: "I have loved thee with an *everlasting* love; therefore with lovingkindness have I drawn thee" (JER. xxxi, 3). That means, God had you in His mind, saw the creature He intended you to be, knew that you would be formed in His own image and after His likeness, and determining to make you for Himself, loved you for what you would be. Oh! it is a wonderful thought, that God's love for each of us had no beginning. It is eternal as Himself. Sin prevents us grasping the idea as we might. When sin came into the world this great Love was thwarted; but it was not destroyed. It was hindered in its development in the creature, but not diminished in the slightest degree. It may seem to have been less than everlasting, but this is only because the breach in God's order caused by sin called into exercise another attribute, namely His Justice. A perfectly just God must have satisfaction for the greatest injury that could be done to His Love. That satisfaction Jesus Christ has given for you, so as to permit the everlasting current of Divine love to run on and fulfil its intention in you. But what did this full, perfect, and sufficient satisfaction and oblation for sin cost? Let St. Paul answer —"He made himself of no reputation, and took upon him the form of a servant, and was made in the likeness of men: And being found in fashion as a man, he humbled himself, and became obedient unto death, even the death of the cross" (PHIL. ii, 7, 8).

(*To be continued.*)

